# Evaluation of treatment of previous cesarean scar pregnancy with methotrexate: a systematic review and meta-analysis

**DOI:** 10.1186/s12958-020-00666-0

**Published:** 2020-11-09

**Authors:** Nader Salari, Mohsen Kazeminia, Shamarina Shohaimi, Anis al-Dawlah Nankali, Masoud Mohammadi

**Affiliations:** 1 Department of Biostatistics, School of Health, Kermanshah University of Medical Sciences, Kermanshah, Iran; 2 Sleep Disorders Research Center, Kermanshah University of Medical Sciences, Kermanshah, Iran; 3 Student Research Committee, Kermanshah University of Medical Sciences, Kermanshah, Iran; 4 Department of Biology, Faculty of Science, Universiti Putra Malaysia, Serdang, Selangor Malaysia; 5 Department of Obstetrics and Gynecology, School of Medicine, Kermanshah University of Medical Sciences, Kermanshah, Iran; 6 Department of Nursing, School of Nursing and Midwifery, Kermanshah University of Medical Sciences, Kermanshah, Iran

**Keywords:** Caesarean scar pregnancy, CSP, MTX, Methotrexate, Meta-analysis

## Abstract

**Background:**

Previous caesarean scar pregnancy is one type of ectopic pregnancy in myometrium and fibrous tissue of previous caesarean scar. One of the therapeutic methods of this type of ectopic pregnancy is treatment with methotrexate. Given various findings on the treatment of caesarean scar pregnancy with methotrexate and lack of global report in this regard, we aimed to achieve a global report on the treatment of CSP with methotrexate through related literature review and analysis of the results of the studies, to enable more precise planning to reduce complications of CSP.

**Method:**

This review study extracted information through searching national and international databases of SID،, Embase, ScienceDirect, ، Scopus, ، PubMed, Web of Science (ISI) between 2003 and January 2020. To perform the meta-analysis, random-effects model and heterogeneity of the studies with I^2^ index were investigated. Data were sanalysed using Comprehensive Meta-Analysis version 2.

**Results:**

In total, 26 articles with a sample size of 600 individuals were enrolled in the meta-analysis. According to the results of the study, the mean level of β-hCG was 28,744.98 ± 4425.1 mIU/ml before the intervention and was 23,836.78 ± 4533.1 mIU/ml after the intervention. The mean intraoperative blood loss (ml) was 4.8 ± 3.76 ml, mean hospital stay (days) was 11.7 ± 1.2 days, mean time for serum-hCG normalization (days) was 41.6 ± 3.2 days, success was 90.7% (95% CI: 86.7–93.5%), and complication was 9% (95% CI: 6.3–12.8%).

**Conclusion:**

The results of the current study show methotrexate significantly reduces β-hCG levels and can be effective in treating caesarean scar pregnancy and its complications.

## Background

Ectopic pregnancy refers to pregnancies which occur out of uterus cavity and 98% of cases occur in the fallopian tube while another 2% occur in uncommon places such as the ovary, abdomen, caesarean scar and other [1]. The prevalence of ectopic pregnancies has increased from 0.5% in the last four decades to 2% in recent decades [1–3]. The rRupture of ectopic pregnancies leads to a high rate of mortality in mothers [4].

Ectopic pregnancy can also cause life-threatening bleeding which needs immediate medical care.

An ectopic pregnancy usually occurs within the first few weeks of pregnancy. Early signs of an ectopic pregnancy include light vaginal bleeding and pelvic pain, upset stomach and vomiting, sharp abdominal cramps, pain on one side of your body, dizziness or weakness, pain in the shoulders, neck, or rectum [1–4].

However, diagnostic methods such as intrauterine sonography, measuring β-hCG level provide context to implement early therapeutic interventions through considerably reducing prevalence of rupture in ectopic pregnancies and mortality cases [2].

Caesarean scar pregnancy (CSP) is one type of ectopic pregnancies in myometrium and fibrous texture of the previous caesarean scar. The reported rate varies from 1 per 1800 to 1 per 2216 pregnancies. The rate of ectopic pregnancies with one to two caesareans is 6.1% [5, 6].

Factors which affect the selection of therapeutic method include gestational age, the tendency for future pregnancy, and present facilities, and therapeutic options such as medicinal treatment, or laparoscopy surgery and laparotomy, sembolisation of uterus artery, injection of potassium chloride, and intra-gestational sac methotrexate [7].

Pharmaceutical treatment is preferred by patients rather than surgery and is an appropriate substitute for surgical therapy because it also reduces treatment costs [7].

Pharmaceutical therapeutic method used mostly in the treatment of ectopic pregnancies is using a single dose of methotrexate and redosing if needed [8].

Methotrexate is an inhibitor of folic acid synthesis, and the inhibition of new purines and pyrimidines leads to the disturbing synthesis of DNA and cell proliferation. These effects appear especially in tissues with high cell turnover, such as pregnancy products [9].

However, various studies have reported between 22 and 48% of failure in the treatment process for this therapeutic regimen [10–14].

Improvement of the efficacy of treatment can prevent serious risks such as the rupture of tubes and intra-abdominal bleeding which may lead to failure in treatment and subsequently a decline in the number of surgeries, days of hospitalisation, and cost of treatment, Two approaches can be considered;firstly, pharmaceutical treatment at a low serum level of β-hCG, and progesterone with a small size of gestational sac without heart rate can be initiated. The second approach is an increase in the efficacy of methotrexate regimen and using a pharmaceutical combination of methotrexate with other medication [9].

Given various findings on the treatment of caesarean scar pregnancy with methotrexate and lack of global report in this regard, we aimed to achieve a global report on the treatment of CSP with methotrexate through related literature review and analysis of the results of the studies, to enable more precise planning to reduce complications of CSP.

## Methods

This systematic review and meta-analysis investigated the treatment of CSP with methotrexate based on studies conducted from 2003 to January 2020. Thereby, published articles in national and international databases of SID، Embase، ScienceDirect، Scopus، PubMed, and Web of Science (ISI) by keywords of cesarean scar pregnancy, CSP, Methotrexate, and MTX were reviewed.

In this study, the AND/OR operators were used to provide more comprehensive access to all articles. Therefore, the AND/OR operator was used to check the common names for the disorder by matching words in the MeSH browser.

((((((((((((((((((Pregnancy [Title/Abstract]) OR (Maternal-Fetal Relations [Title/Abstract])) OR (Pregnant Women [Title/Abstract])) OR (Pseudopregnancy [Title/Abstract])) OR (Prenatal Care [Title/Abstract])) AND (cesarean [Title/Abstract])) AND (scar [Title/Abstract])) OR (Cicatrix [Title/Abstract])) OR (Scarring [Title/Abstract])) OR (Scars [Title/Abstract])) OR (CSP [Title/Abstract])) AND (Methotrexate [Title/Abstract])) OR (Amethopterin [Title/Abstract])) OR (Methotrexate Hydrate [Title/Abstract])) OR (Methotrexate Sodium [Title/Abstract])) OR (Methotrexate, (D)-Isomer [Title/Abstract])) OR (Methotrexate, Sodium Salt [Title/Abstract])) OR (Methotrexate, (DL)-Isomer [Title/Abstract])) OR (MTX [Title/Abstract]))))))))

The inclusion criteria to select articles were as follow observational studies (non-interventional studies) and accessibility of the full text. In order to obtain more information, references for the relevant articles were reviewed to access to other articles.

### Selection of articles

At first, all the articles on the treatment of CSP with methotrexate were gathered by researchers and the eligible articles were included based on the inclusion and exclusion criteria. Exclusion criteria include irrelevant articles, duplicates, ambiguity in materials and methods, and lack of access to full-texts.

To control for bias, the literature review was performed by two independent researchers, and in case of disagreement, the article was referred to the supervisor to be reviewed. Finally, 35 articles were entered in the third phase of qualitative assessment.

Articles derived from observational studies were included while review, case-control, cohort, and interventional studies were excluded from the list of articles. Duplicate publications and multiple publications from the same population were removed using citation management software EndNote (version X7, for Windows, Thomson Reuters).

### Qualitative assessments of articles

The CONSORT checklist was used to evaluate the quality of articles. This checklist consists of the design, background, literature review, location and time of study, outcome, inclusion criteria, sample size and statistical analysis. Articles with scores in 6–7 items were considered as high-quality articles while articles which had between two to seven items and two items were considered as articles with moderate and low quality, respectively [15].

In the current study, 26 high and moderate-quality articles were entered in the systematic review and meta-analysis while 9 low-quality articles were excluded.

### Data extraction

All the final articles entered in the meta-analysis were extracted using a pre-prepared check-list. The checklist includes article title, first author, year of publication, study location, sample size, the mean level of β-hCG, intraoperative blood loss (ml), hospital stay (days), time for serum-hCG snormalisation (days), success percentage, complication percentage and methods.

### Data analysis

To assess heterogeneity of enrolled studies, the I^2^ index was used, and meta regression analysis was used to investigate the association between mean level of β-hCG, year of publication and sample size and the probability of publication bias in results was measured using the funnel plot, the Egger test and the significance level of 0.05. To assess the effect of each study individually on the final outcome, sensitivity test was used. Analysis of data was performed using Comprehensive Meta-Analysis Software (Version 2).

## Results

**Fig. 1:**
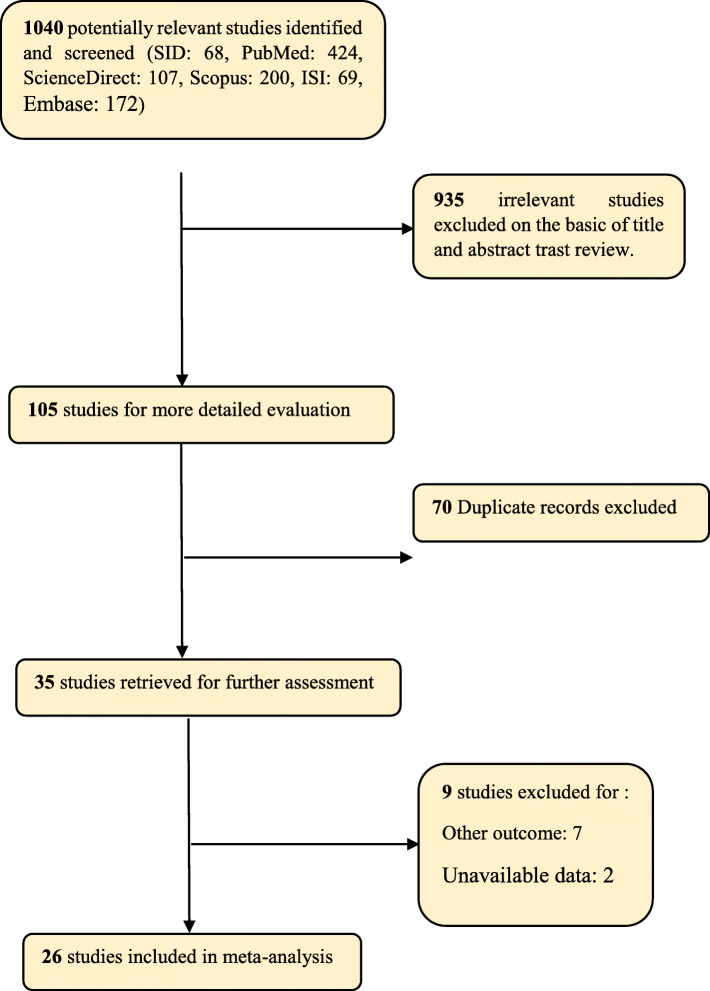
Flow diagram of study selection

In this study, all studies on the treatment of CSP with methotrexate without time limitation and based on PRISMA guideline were assessed systematically. In primary searching, 1040 articles were identified, which finally, 26 articles published between 2003 and January 2020 were entered in the final analysis (Fig. 1) (Table 1).

**Table 1:** Characteristics of meta-analysis studies

Author, year, Reference	Country	Sample size	β-hCG level (mIU/ml)	Methods of intervention	Characteristic	Quality
Wang-1, 2018, [16]	China	46	Before = 44,603.28 After = 37,712.91	50 mg/m^2^ intramuscularly two times (Day 0 and Day 4) MTX	Age (years) = 31.15 ± 5.59 Intraoperative blood loss (ml) = 55.33 ± 44.19 Hospital stay (days) = 9.59 ± 2.46 Gestational age (day) = 48.96 ± 8.27 Number of cesarean deliveries = 1.17 ± 0.383 Fetal heart beat positive, *n* = 21	High
Wang-2, 2018, [16]	China	14	Before = 25,648.86 After = 31,005.21	intravenously for 5 days (Day 0 to Day 5) MTX	Age (years) = 34.14 ± 4.86 Intraoperative blood loss (ml) = 42.86 ± 22.68 Hospital stay (days) = 13.86 ± 3.88 Gestational age (day) = 52.15 ± 7.94 Number of cesarean deliveries = 1.57 ± 0.514 Fetal heart beat positive, *n* = 5	High
Giampaolino, 2018, [17]	Italy	19	Before = 1790 After = 35,034	50 mg/m^2^ intramuscularly One times MTX+ D&C	Age (years) = 32.68 ± 3.92 Gestational age (day) = 50.47 ± 4.43 Complication, *n* = 0	High
Jiang, 2011, [18]	China	45	After = 28,717	50 mg/m^2^ intramuscularly One times MTX	Age (years) = 34.46 ± 5.19 Intraoperative blood loss (ml) = 706.89 ± 642.08 Time for serum-hCG normalization (days) = 20.62 ± 5.41 Complication, *n* = 3 Success% = 93.3	High
Shen, 2012, [19]	China	46		intravascularly One times MTX	Age (years) = 32.7 ± 6.0 Hospital stay (days) = 10.5 ± 1.0 Time for serum-hCG normalization (days) = 37.7 ± 4.8 Time for CSP mass disappearance (days) = 33.3 ± 4.3 Gestational age (day) = 55.5 ± 2.4 Complication, *n* = 1 Success% = 97.8	High
Qi, 2015, [20]	China	22	After = 45,710	UAE + MTX + 50 mg/m^2^ Intramuscular	Intraoperative blood loss (ml) = 80.25 ± 113.92 Time for serum-hCG normalization (days) = 31.18 ± 14.80 Gestational age (day) = 59.86 ± 17.67 Complication, *n* = 4 Success% = 77.3	Medium
Gao, 2014, [21]	China	119	Before = 45,321.50 After = 43,586	D&C + MTX + 50 mg/m^2^ Intramuscular	Intraoperative blood loss (ml) = 261.0 ± 357.4 Hospital stay (days) = 14.6 ± 9.2 Time for serum-hCG normalization (days) = 40.5 ± 17.2 Gestational age (day) = 48.4 ± 7.6 Number of cesarean deliveries = 1.2 ± 0.4 Fetal heart beat positive, *n* = 8 Complication, *n* = 10 Success% = 91.6 Gravidity = 3.9 ± 1.6 Parity = 1.4 ± 0.6	High
Liu, 2016, [22]	China	26	Before = 8242 After=	MTX-curettage + Intramuscular (50 mg/m^2^ body surface area)	Age (years) = 31.82 ± 4.80 Intraoperative blood loss (ml) = 335 Hospital stay (days) = 19.38 Time for serum-hCG normalization (days) = 56.15 ± 15.99 Gestational age (day) = 48.4 ± 7.6 Fetal heart beat positive, *n* = 8 Complication, *n* = 4 Success% = 84.6	High
Cao, 2018, [23]	China	36		UAE + MTX + 40 mg/m^2^ Intramuscular	Age (years) = 33.46 ± 4.47 Intraoperative blood loss (ml) = 11.44 ± 4.87 Hospital stay (days) = 5.39 ± 1.02 Time for serum-hCG normalization (days) = 34 Complication, *n* = 3 Success% = 91.7	High
Feng, 2016, [24]	China	11		UAE + MTX + 50 mg/m^2^ Intramuscular	Age (years) = 32.20 ± 4.83 Intraoperative blood loss (ml) = 16 ± 3.8 Time for serum-hCG normalization (days) = 27 Complication, *n* = 0 Success% = 95.8	High
Sevket, 2014, [25]	Turkey	11		MTX-curettage + Intramuscular	Hospital stay (days) = 14.45 ± 4.96 Complication, *n* = 0 Success% = 95.8	Medium
Fadhlaoui, 2012, [26]	Tunisia	1		50 mg/m^2^ intramuscularly two times (Day 0 and Day 4) MTX	Age (years) = 35 Hospital stay (days) = 8 Time for serum-hCG normalization (days) = 34 Complication, *n* = 0	Medium
Wang, 2009, [27]	China	21	After = 13,576	D&C + MTX + Intramuscular	Age (years) = 33.4 ± 4.8 Time for serum-hCG normalization (days) = 38 Complication, *n* = 5 Success% = 76.2	High
Abdelazim, 2017, [28]	Kazakhstan	1		Multi-dose MTX+ Intramuscular	Age (years) = 37 Hospital stay (days) = 4 Fetal heart beat positive, *n* = 8 Complication, n = 0	High
Uludag-1, 2016, [29]	Turkey	17	Before = 27,970 After = 11,010	local methotrexate injection	Age (years) = 32.76 ± 5.25 Hospital stay (days) = 7.05 ± 2.77 Complication, *n* = 0 Success% = 97.2	High
Uludag-2, 2016, [29]	Turkey	27	Before = 7606 After = 4725	systemic methotrexate	Age (years) = 31.07 ± 4.17 Hospital stay (days) = 11.96 ± 4.02 Complication, *n* = 0 Success% = 98.2	High
Ko, 2015, [30]	China	10	Before = 50,666	intralesional methotrexate	Age (years) = 34.9 ± 4.8 Complication, *n* = 2 Success% = 80.0	High
Yin, 2014, [31]	China	22	Before = 40,154.17 After = 2531.56	intramuscularly two times (Day 0 and Day 4) MTX	Age (years) = 28.5 ± 3.9 Intraoperative blood loss (ml) = 139 ± 4.83 Hospital stay (days) = 25 ± 6.61 Gestational age (day) = 56.14 ± 21.12 Complication, *n* = 1 Success% = 95.5	High
Cok, 2015, [32]	Turkey	18	Before = 12,699	local methotrexate injection	Age (years) = 33.7 ± 3.4 Complication, *n* = 3 Success% = 83.3	High
Timor-Tritsch, 2015, [33]	USA	33		MTX + 50 mg/m^2^ Intramuscular	Complication, *n* = 1 Success% = 93.9	Medium
Yamaguchi, 2014, [34]	Japan	8	Before = 45,823	local MTX injection	Age (years) = 32.3 ± 4.1 Time for serum-hCG normalization (days) = 78.5 Complication, *n* = 0 Success% = 94.4	High
Seow, 2013, [35]	China	11	Before = 20,520 After = 22,500	One injection of Intramuscular MTX	Age (years) = 33.8 ± 4.0 Time for serum-hCG normalization (days) = 48 Gestational age (day) = 35 ± 21 Complication, *n* = 0 Success% = 95.8	High
Li, 2012, [36]	China	28	Before = 26,426 After=	low-dose methotrexate-curettage + Intramuscular	Age (years) = 31.2 ± 2.2 Complication, *n* = 0 Success% = 96.2	High
Jurkovic, 2003, [37]	UK	6	Before = 36,388 After = 25,000	local injection of 25 mg/m^2^ methotrexate	Age (years) = 39.4 ± 3.8 Complication, *n* = 2 Success% = 66.7	High
Pirjani −1, 2015, [38]	Iran	1		Local MTX + Systemic MTX	Age (years) = 35 Time for serum-hCG normalization (days) = 56 Gestational age (day) = 84 Fetal heart beat positive, *n* = 0 Complication, *n* = 0 Success% = 92.9	Medium
Pirjani −2, 2015, [38]	Iran	1		Systemic MTX + Local MTX	Age (years) = 37 Time for serum-hCG normalization (days) = 35 Gestational age (day) = 35 Fetal heart beat positive, *n* = 1 Complication, *n* = 0 Success% = 92.9	Medium

### Publication bias

**Fig. 2:**
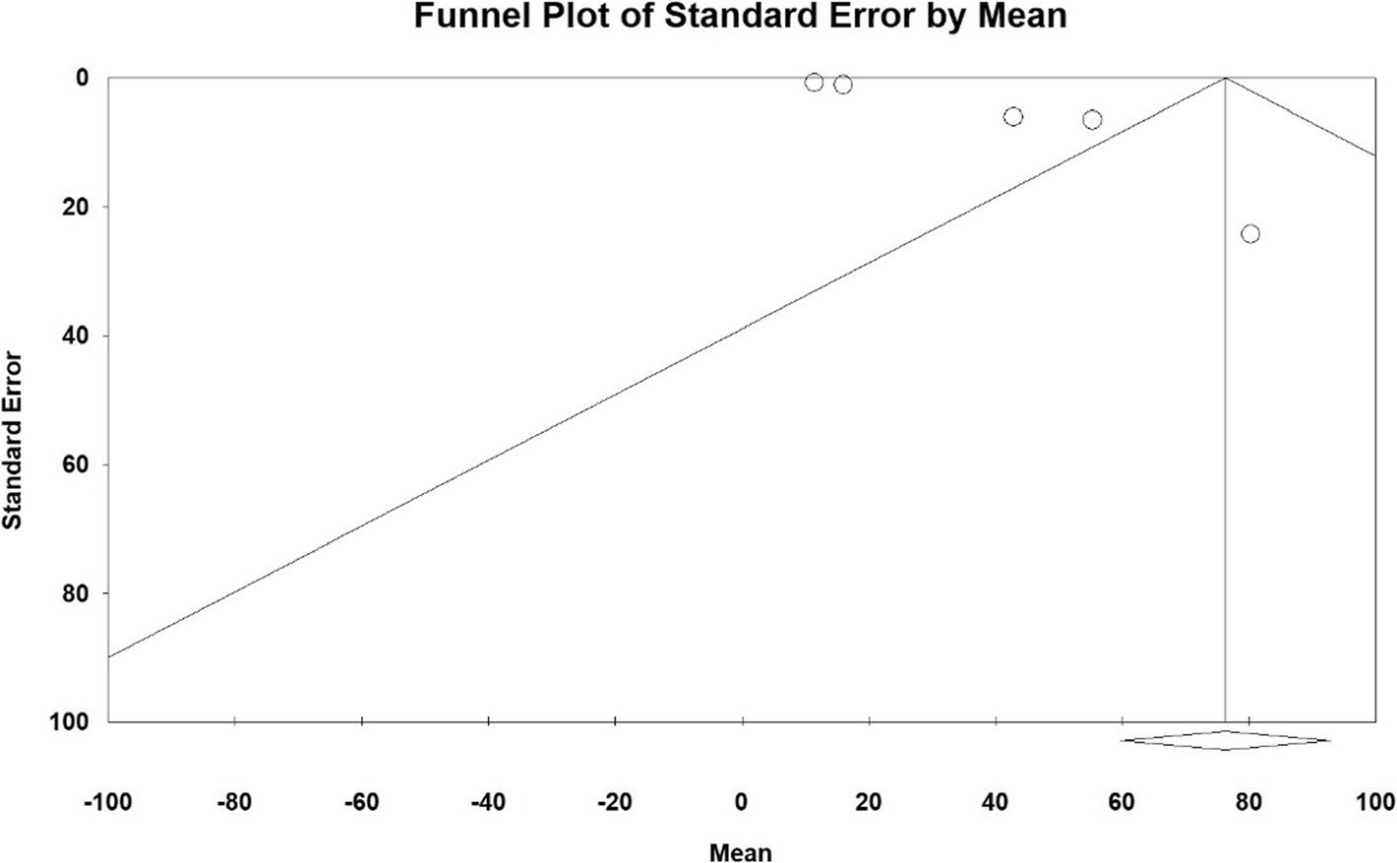
Funnel plot of results related to Intraoperative blood loss (ml)

The publication bias in results of intraoperative blood loss (ml) by funnel plot and Egger test at significance level of 0.05 indicate the lack of bias in publication in the current study (*P* = 0.06) (Fig. 2).

**Fig. 3:**
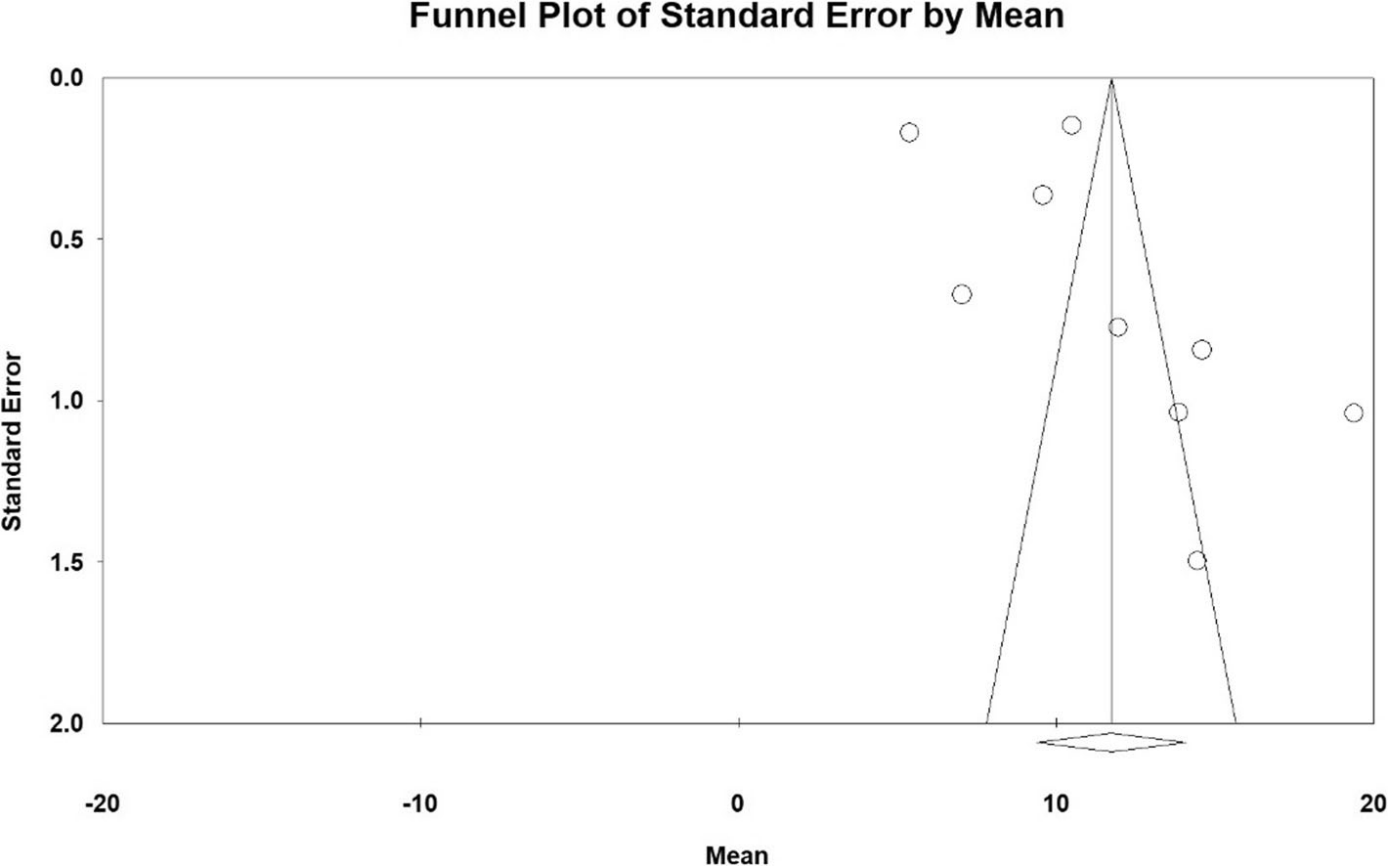
Funnel plot of results related to Hospital stay (days)

The publication bias in results of hospital stay (days) by funnel plot and Egger test at significance level of 0.05 indicates lack of bias in publication in the current study (*P* = 0.269) (Fig. 3).

**Fig. 4:**
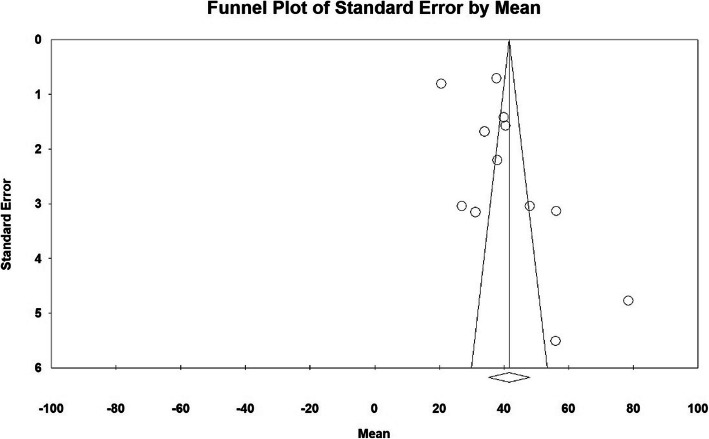
Funnel plot of results related to Time for serum-hCG snormalisation (days)

The publication bias in results of time for serum-hCG snormalisation (days) by funnel plot and Egger test at significance level of 0.05 indicates lack of bias in publication in the current study (*P* = 0.095) (Fig. 4).

**Fig. 5:**
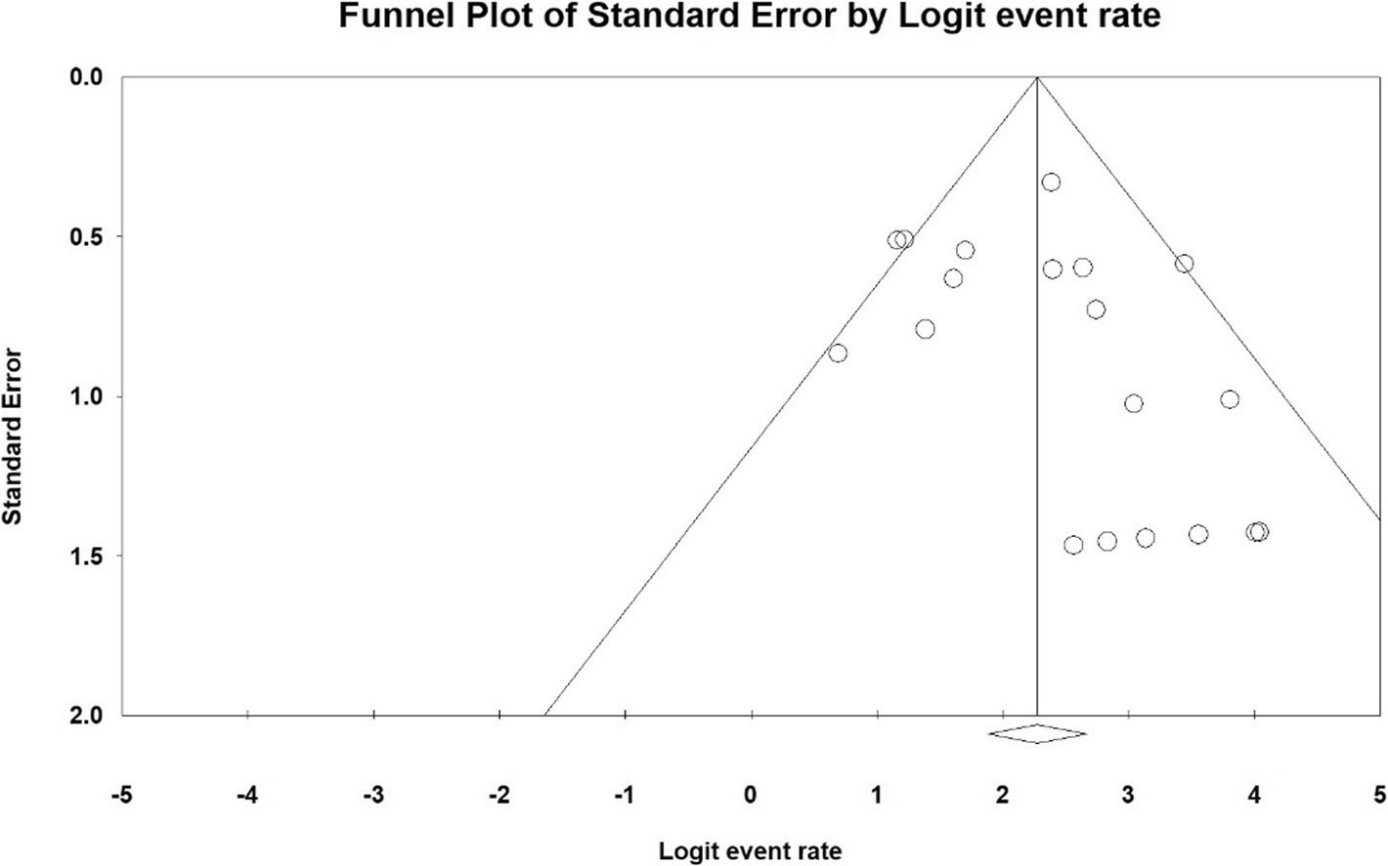
Funnel plot of results related to percentage of success

The publication bias in results of success by funnel plot and Egger test at significance level of 0.05 indicates lack of bias in publication in the current study (*P* = 0.082) (Fig. 5).

**Fig. 6:**
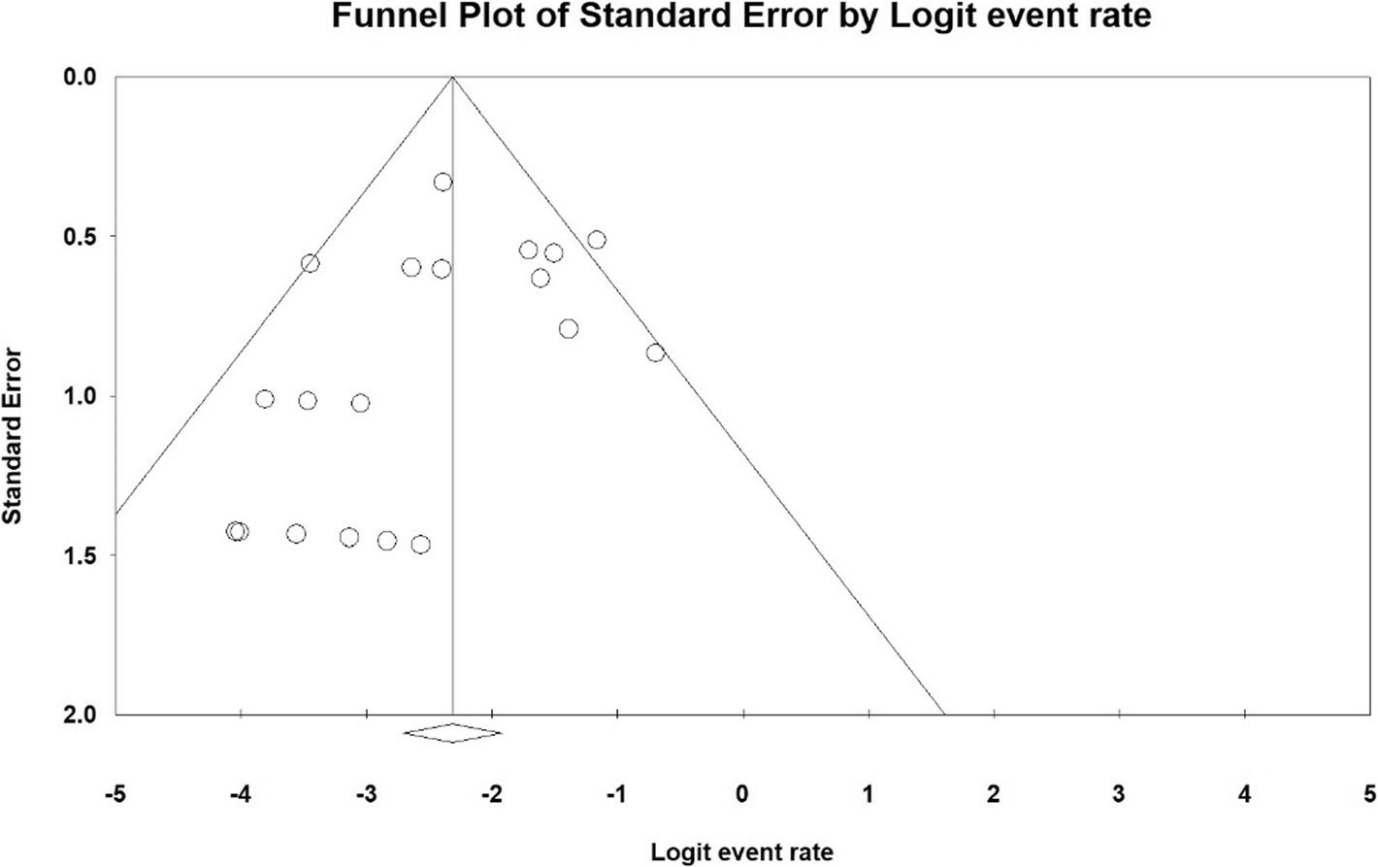
Funnel plot of results related to percentage of complication

The publication bias in results of complication by funnel plot and Egger test at significance level of 0.05 indicates lack of bias in publication in the current study (*P* = 0.07) (Fig. 6).

### Heterogeneity

According to results obtained from the test (I^2^: 100), (I^2^: 100), (I^2^: 98.2), (I^2^: 98), (I^2^: 97.8), and (I^2^: 29.7) and due to the heterogeneity of selected studies, random effect model to combine studies and a common estimate of the mean level of β-hCG before and after the intervention, intraoperative blood loss (ml), hHospital stay (days), time for serum-hCG snormalisation (days), success percentage, and complication percentage were used.

The total sample size was 600 individuals and the characteristics of selected articles are presented in Table 1.

Out of 26 articles entered the meta-analysis, 17 articles were for methotrexate alone, 3 articles were on MTX + D&C, three articles were on UAE + MTX, and three articles were on MTX-curettage.

**Fig. 7:**
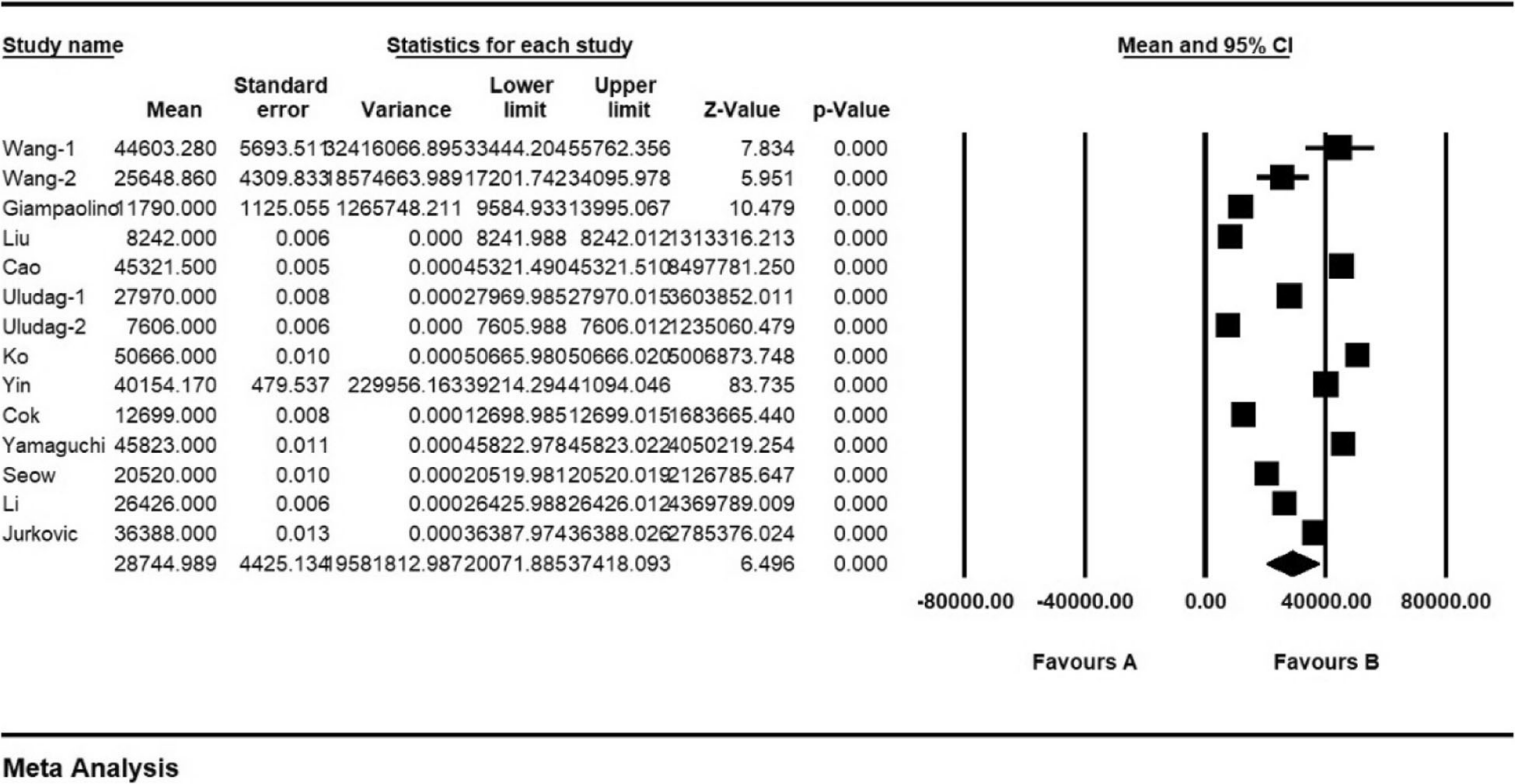
mean level of β-hCG before the intervention and confidence interval of 95%. The middle point of each line shows mean level of β-hCG before the intervention in each study, and the rhombic figure shows mean level of β-hCG before intervention for all the studies

**Fig. 8:**
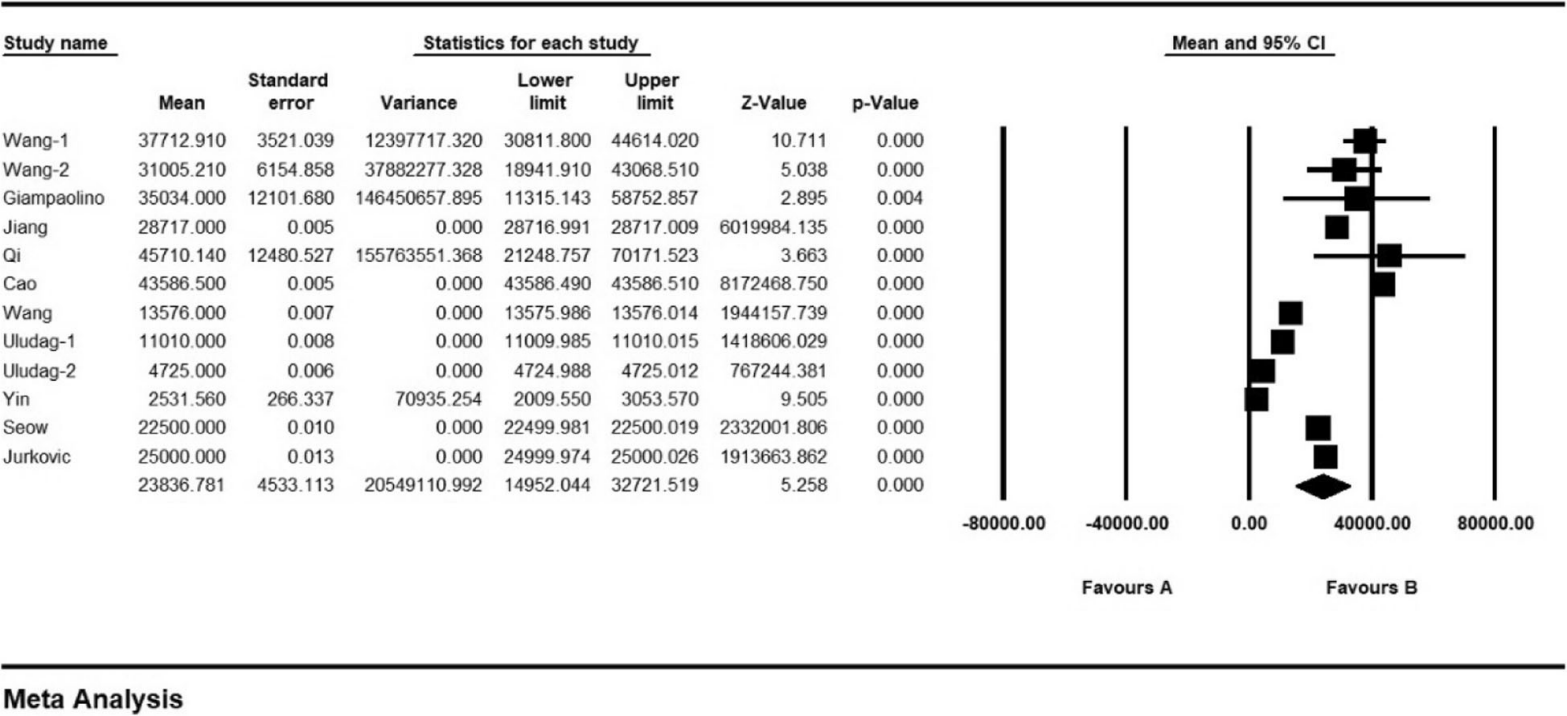
Mean level of β-hCG after the intervention and confidence interval of 95%. The middle point of each line shows mean level of β-hCG before the intervention in each study, and the rhombic figure shows mean level of β-hCG before intervention for all the studies

According to results of the study, the mean level of β-hCG was 28,744.98 ± 4425.1 mIU/ml before the intervention and 23,836.78 ± 4533.1 mIU/ml after the intervention, which shows the reducing effect of the drug on patients (Figs. 7 and 8).

**Fig. 9:**
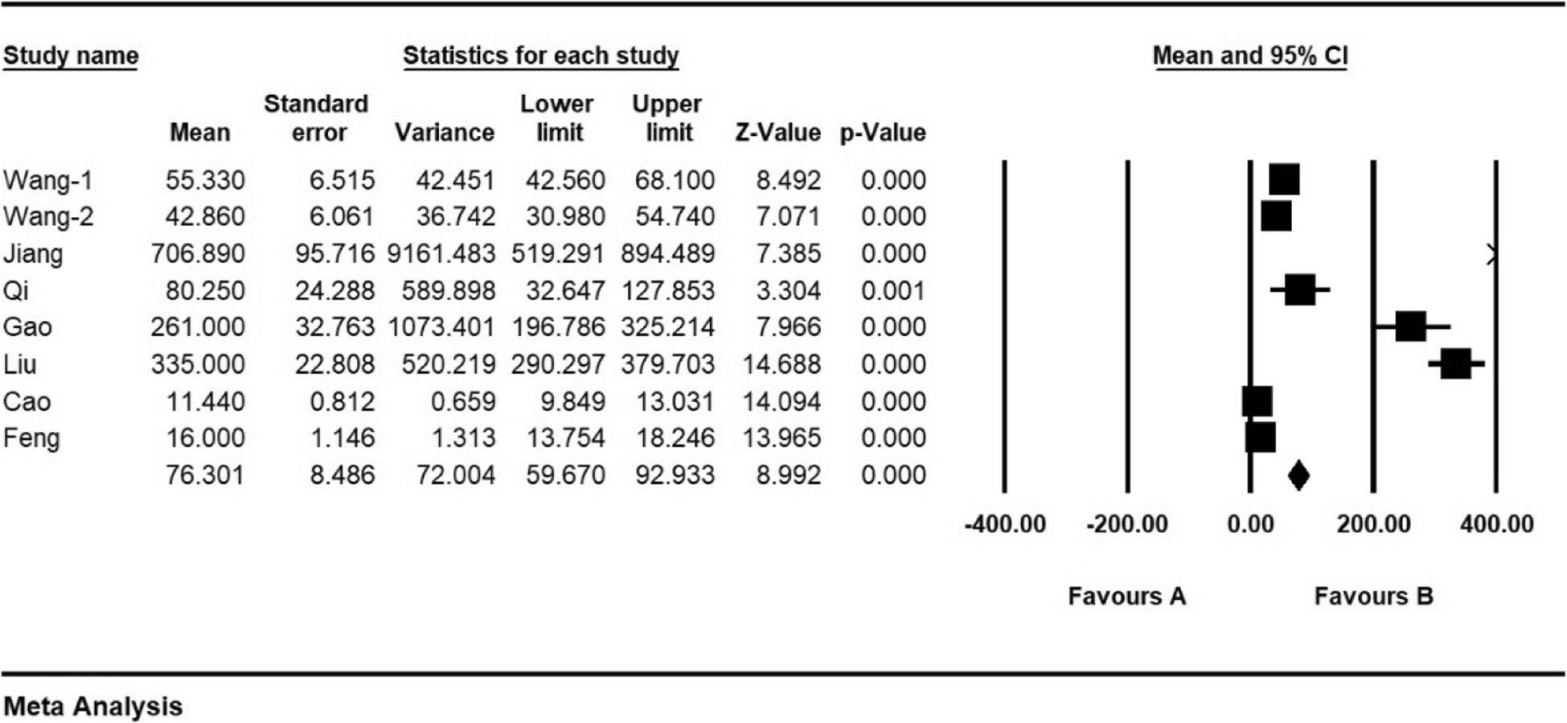
Mean Intraoperative blood loss (ml) and confidence interval of 95%. The middle point of each line shows mean Intraoperative blood loss (ml) in each study, and the rhombic figure shows mean Intraoperative blood loss (ml) for all the studies

According to the results of the study, mean intraoperative blood loss (ml) was 76.3 ± 8.4 ml (Fig. 9).

**Fig. 10:**
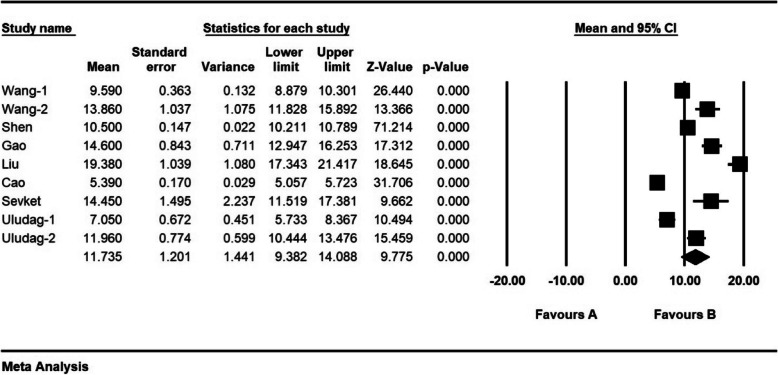
Mean Hospital stay (days) and confidence interval of 95%. The middle point of each line shows mean Hospital stay (days) in each study, and the rhombic figure shows mean Hospital stay (days) for all the studies

According to the results of the study, mean hospital stay (days) was 11.7 ± 1.2 days (Fig. 10).

**Fig. 11:**
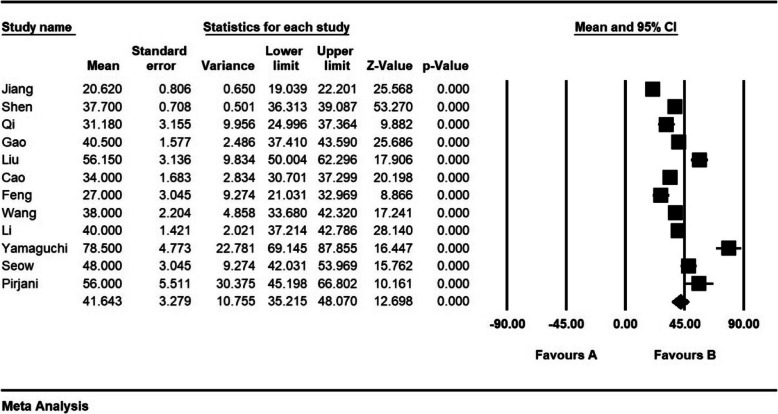
Time for serum-hCG snormalisation (days) and confidence interval of 95%. The middle point of each line shows Time for serum-hCG snormalisation (days) in each study, and the rhombic figure shows Time for serum-hCG snormalisation (days) for all the studies

According to the results of the study, time for serum-hCG snormalisation (days) was 41.6 ± 3.2 days (Fig. 11).

**Fig. 12:**
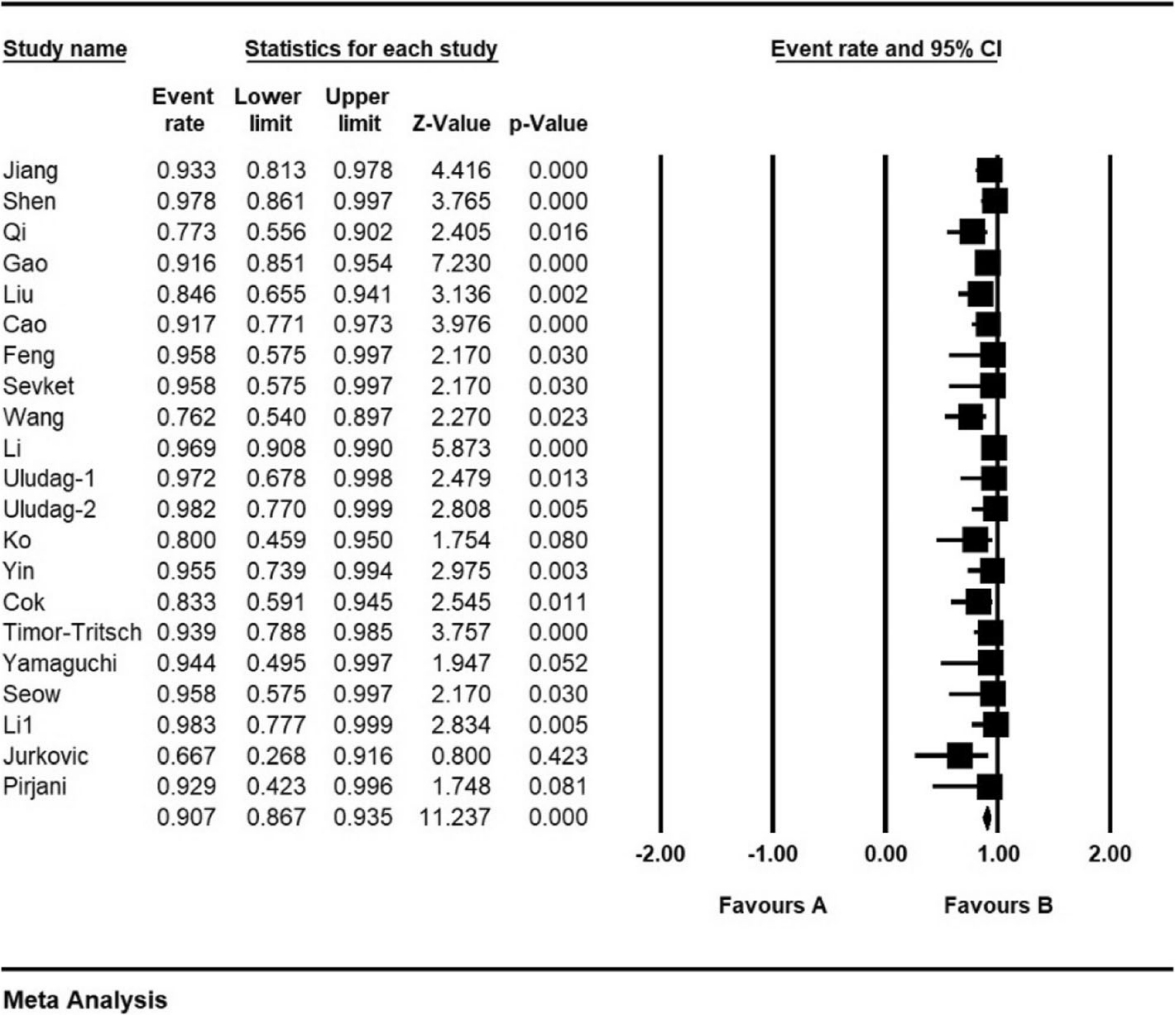
Success and confidence interval of 95%. The middle point of each line shows Success in each study, and the rhombic figure shows Success for all the studies

According to the results of the study, success percentage was 90.7% (95% CI: 86.7–93.5%) (Fig. 12).

**Fig. 13:**
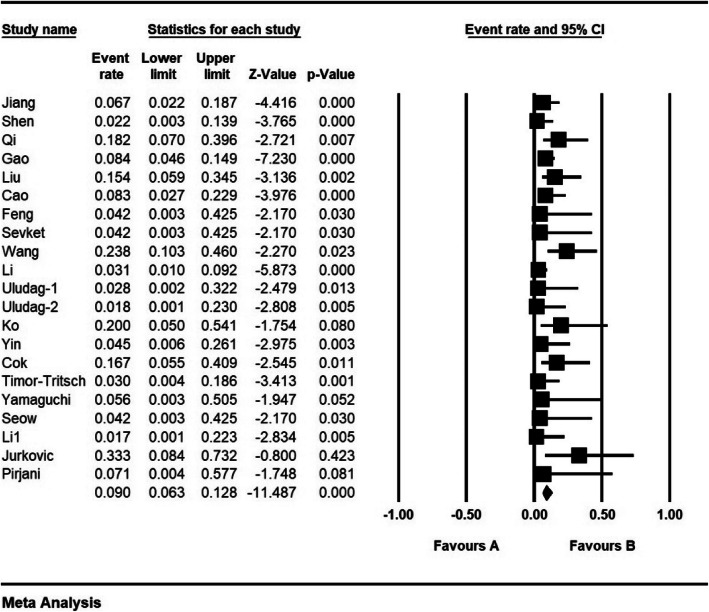
Complication and confidence interval of 95%. The middle point of each line shows Complication in each study, and the rhombic figure shows Complication for all the studies

According to the results of the study, complication percentage was 9% (95% CI: 6.3–12.8%) (Fig. 13).

## Discussion

This study aimed to determine the treatment of CSP with methotrexate through a systematic review and meta-analysis.

According to findings of this study, mean level of β-hCG before the intervention was 28,744.98 ± 4425.1 mIU/ml and was 23,836.78 ± 4533.1 mIU/ml after the intervention, which indicates methotrexate considerably decreases the β-hCG level.

In addition, time for serum-hCG snormalisation was 41.6 ± 3.2 days. Methotrexate, a folic acid antagonist by deactivating dihydrofolate reductase enzyme, depletes the available reservoir of tetrahydrofolate, and tissues with high-turnover such as trophoblasts are particularly vulnerable to this medication. Treatment with methotrexate reduces the speed of increasing β-hCG [39]. Single-dose, double dose and multidose therapeutic regimens of methotrexate are recommended.

For single-dose regimen, 50 mg methotrexate per m^2^ of body and the level of β-hCG at days of 4 and 7 are measured, and should be decreased 15% or more; this follow-up is done weekly. In most women, the concentration of β-hCG naturally increases between days 1 and 4, but, thereafter hormone level decreases. Each subsequent increase between days 4 and 7 is an indication for prescribing second dose (day 7) and is a determinant of decrease response of hormone level at day 11. In case of failure in medicinal treatment, surgery is recommended [40].

In double dose regimen, methotrexate is prescribed in days of 1 and 4 and serum level of β-hCG in days of 4–7 is measured. If the value decreases less than 15%, the third dose should be prescribed and is assessed on day 11. If needed, the fourth dose can also be prescribed, and surgery is recommended if no response is given [41].

For multi-dose regimen, methotrexate (1 mg/kg of muscular bodyweight) up to four doses are injected every other day to induce 15% or more decrease in the concentration of β-hCG and the level of hormone at days 1, 3, 5 and 7 should be checked.

In case of lack of appropriate decline, surgery is recommended [41]. Soliman et al. (2006) found that in cases of β-hCG higher than 3000 to 4000, the probability of surgery and failure of medicinal therapy is greater [42]. In the study by Lipscomb et al. (2005), level of β-hCG before the treatment is the most important factor in the failure of treatment [43].

The study by Eskandar (2007) reported that level of β-hCG as low as 2000 mlu/ml is a predictor of failure in medicinal treatment with a single dose of methotrexate [44].

The β-hCG level is an important predictor in the diagnosis of ectopic pregnancy and follow-up of response to treatment in patients. Mol reported that if β-hCG is less than 1500 IU/Liter, it is better to use single-dose regimen, but if β-hCG is less than 3000 IU/Liter, multi-dose regimen is better [45]. In two independent studies by Gabbur and Erdem on patients who underwent a single-dose regimen of MTX, β-hCG level in the first day in a successful day is less than the group which needs second or more dose [46, 47].

Although, it was evident that by an increase in β-hCG, the probability of successful treatment decreases, there is no stable distinct level below which the treatment is more successful and the upper level to be relative inhibition for treatment [48]. In the study by Erdem, three out of 34 patients with failure in treatment had β-hCG greater than 4000 mlu/ml and two cases were with cardiac activity [47]. Menon showed on 503 patients that when β-hCG is greater than 5000, the failure in treatment increases, and therefore, it is better to use MTX in these patients cautiously [48].

Markwitz showed that for 68 patients, when β-hCG is 1790 mlu/ml, there is a risk of failure in treatment [49], and in the study by Gamzu, there was a 97% success in the treatment of β-hCG less than 2000 in comparison to 74% of success in the treatment of β-hCG higher than 2000 [50].

Since, in the current systematic review and meta-analysis, MTX leads to more than 15% reduction (17%) in β-hCG level; therefore it can be considered as one successful pharmaceutical treatment in CSP. According to the findings of the current systematic review and meta-analysis, the success percentage was 90%, and mean hospital stay (days) was also 11.7 ± 1.2 days. The rate of success in medicinal therapy of ectopic pregnancy by MTX is diverse, and about 71–100% [51].

In a research conducted in Iran (2000), out of 72 patients with ectopic pregnancy, 4 cases (5.6%) were treated medicinally, which only one of them was successful, and three other patients need surgery [52], which was inconsistent with this study.

Its reason might be lack of probable follow-up of the patient and the rapid decision on performing surgery. In a retrospective study in Australia, out of 637 women diagnosed for ectopic pregnancy, 74 patients underwent medicinal treatment with MTX, which 14 cases were with failure (18.9%), and out of 537 patients who underwent surgery, 30 patients (5.6%) need reoperation [53]. From the year 1996 to 2001 in the USA, out of 1327 patients treated with MTX, 1181 patients (89%) were treated successfully.

In a study in France (2003) on 137 women with non-ruptured ectopic pregnancy, 70 patients received MTX intramuscularly, and 67 patients received injection intra-hematosaplinx under sonographic control. Rate of success was 79.6%, and topical use of MTX increases the probability of of success considerably [54].

In the study by Lewis et al. on 119 patients underwent medicinal therapy with MTX, 70% of patients received a single dose, and 11% received double dose, which rate of success was reported as 79% [55], which were in line with the current study. According to the results of the current systematic review and meta-analysis, complication achieved 9%. MTX is with numerous side effects and in some cases is life-threatening. Gastrointestinal complications commonly occur by MTX, and renal toxification caused by the deposit of medication in renal tubules (particularly in acidic urine, patients with decreased volume and high serum level of MTX), and glumerol might occur. In addition, MTX by contraction of afferent artery and contraction of mesangial cells causes renal failure [56].

Hematologic complications, hepatic toxification, and pulmonary toxification are of other complications of MTX. Acute increase in serum transaminases from two to 20 times greater than standard value was observed in 80% of patients which is relieved in one to two weeks spontaneously. If the value of alanine transferase does not reach to less than 180 IU/L at the initiation of following treatment course, a subsequent dose of MTX must be reduced or delayed [57].

Finally, due to current systematic review and meta-analysis and similar studies, it seems that treatment of CSP with MTX is an effective, low-risk, and low-cost method, and by considering this tip that most of the patients with CSP tend to preserve their fertility potential in future, it should be tried to treat these patients with a medicinal method.

Therefore, it is recommended that there should be a change in attitude and behaviour of co-workers to face patients with ectopic pregnancy. First, in order to early diagnosis and second the application of medicinal therapy in most suitable cases of surgical treatment, and surgical treatment should be done only in cases of impossible medicinal therapy or in lack of access to follow the patient.

## Conclusion

The results of the current study show that methotrexate significantly reduces β-hCG levels and can be effective in treating caesarean scar pregnancy and its complications.

## Data Availability

Datasets are available through the corresponding author upon reasonable request.

## Abbreviations

SID: Scientific Information Database
CONSORT: Consolidated Standards of Reporting Trials
PRISMA: Preferred Reporting Items for Systematic Reviews and Meta-Analysis
